# Urinary podocyte stress marker as a prognostic indicator for diabetic kidney disease

**DOI:** 10.1186/s12882-024-03471-8

**Published:** 2024-01-24

**Authors:** Lingfeng Zeng, Jack Kit-Chung Ng, Winston Wing-Shing Fung, Gordon Chun-Kau Chan, Kai-Ming Chow, Cheuk-Chun Szeto

**Affiliations:** 1 Department of General Medicine, The Xiangya Second Hospital of Central South University, Changsha, China; 2 Carol & Richard Yu Peritoneal Dialysis Research Centre, Department of Medicine & Therapeutics, Prince of Wales Hospital, Randwick, Australia; 3 Li Ka Shing Institute of Health Sciences (LiHS), Faculty of Medicine, The Chinese University of Hong Kong, Shatin, Hong Kong China

**Keywords:** Podocyte, Proteinuria, Chronic kidney disease, anemia

## Abstract

**Background:**

Diabetic kidney diseases (DKD) is a the most common cause of end-stage kidney disease (ESKD) around the world. Previous studies suggest that urinary podocyte stress biomarker, e.g. podocin:nephrin mRNA ratio, is a surrogate marker of podocyte injury in non-diabetic kidney diseases.

**Method:**

We studied 118 patients with biopsy-proved DKD and 13 non-diabetic controls. Their urinary mRNA levels of nephrin, podocin, and aquaporin-2 (AQP2) were quantified. Renal events, defined as death, dialysis, or 40% reduction in glomerular filtration rate, were determined at 12 months.

**Results:**

Urinary podocin:nephrin mRNA ratio of DKD was significantly higher than the control group (*p* = 0.0019), while urinary nephrin:AQP2 or podocin:AQP2 ratios were not different between groups. In DKD, urinary podocin:nephrin mRNA ratio correlated with the severity of tubulointerstitial fibrosis (*r* = 0.254, *p* = 0.006). and was associated with the renal event-free survival in 12 months (unadjusted hazard ratio [HR], 1.523; 95% confidence interval [CI] 1.157–2.006; *p* = 0.003). After adjusting for clinical and pathological factors, urinary podocin:nephrin mRNA ratio have a trend to predict renal event-free survival (adjusted HR, 1.327; 95%CI 0.980–1.797; *p* = 0.067), but the result did not reach statistical significance.

**Conclusion:**

Urinary podocin:nephrin mRNA ratio has a marginal prognostic value in biopsy-proven DKD. Further validation is required for DKD patients without kidney biopsy.

**Supplementary Information:**

The online version contains supplementary material available at 10.1186/s12882-024-03471-8.

## Background

Diabetic kidney disease (DKD) is the most common cause of end-stage kidney disease (ESKD) worldwide [1]. There is a critical need for effective methods for diagnosing, treating, and monitoring DKD. While estimated glomerular filtration rate (eGFR) and albuminuria are simple and non-invasive, they lack specificity and sensitivity for these purposes [2]. Therefore, there is a pressing need for novel biomarkers to monitor and stratify the risk of DKD.

The podocyte plays a crucial role in maintaining normal glomerular architecture and is a primary focus in many kidney diseases [3, 4]. DKD is characterized by early and severe podocyte involvement [5]. Podocyte injury leads to the release of various podocyte-derived molecules into the urine, making them potential biomarkers for kidney diseases [6]. Measuring the level of urinary podocyte-specific mRNA has been proposed as an alternative method to assess podocyte injury and the severity of podocyte loss in the urine [7]. Traditionally, the urinary levels of podocyte-specific molecules were considered as a surrogate marker of the number of podocyte lost from the glomeruli [8, 9]. More recently, it is recognized that the expression levels of various podocyte-specific molecules are not uniform, and attention has shifted to the urinary level ratios of several podocyte-specific molecules as they may indicate the severity of podocyte stress or sub-lethal podocyte injury [7, 10, 11]. In a rat model, the urinary podocin-to-nephrin mRNA ratio correlated with the extent of histological damage [10]. Additionally, in healthy individuals, the mean arterial pressure correlated with the urinary podocin-to-nephrin mRNA ratio (a marker of podocyte stress) and the urinary podocin-to-aquaporin-2 mRNA ratio, which represent podocyte stress and the relative severity of podocyte injury compared to tubular injury, respectively [11]. Another study found that the severity of glomerular injury specifically correlated with the urinary podocin-to-aquaporin-2 and nephrin-to-aquaporin-2 mRNA ratios [7].

However, the above studies had small sample size and recruited patients with hypertension or acute glomerular disease, and the prognostic role of urinary mRNA indices has not been studied in DKD. In this study, we investigated the role of three urinary mRNA indices as markers of podocyte stress or relative podocyte injury in predicting the disease progression for patients with biopsy-proven DKD.

## Patients and methods

The study received approval from the Clinical Research Ethical Committee of the Chinese University of Hong Kong (approval number CREC-2016.480). All study procedures adhered to the Declaration of Helsinki.

### Subjects

We recruited 118 consecutive patients with type 2 diabetes mellitus and kidney biopsy-proven diabetic nephropathy from our center. As controls, we also studied 13 non-diabetic patients with biopsy-proven hypertensive nephrosclerosis (HTN). On the day of kidney biopsy, we collected a whole-stream early-morning urine sample. Additionally, we reviewed their demographic and clinical data, including serum creatinine and proteinuria. The estimated glomerular filtration rate (eGFR) was calculated using the Chronic Kidney Disease Epidemiology Collaboration (CKD-EPI) equation [12].

### RNA extraction

The method for extracting and quantifying mRNA in urinary sediment has been previously explained [13]. Briefly, after collecting urine samples, they were centrifuged at 4 °C for 15 min at 3200 g. The supernatant was discarded, and the pellet was suspended in 1.5 mL of phosphate buffered saline treated with diethyl pyrocarbonate. The suspension was then centrifuged at 4 °C for 5 min at 12,000 g. The washed pellet was re-suspended in lysis buffer (RNeasy; Qiagen, Germantown, MD, USA) and stored at -80 °C until RNA extraction. The urinary pellet was purified using an RNeasy mini kit (Qiagen), and cDNA was prepared using the SuperScript™ IV First-Strand Synthesis System (ThermoFisher, Germany).

### RNA preparation and RT-qPCR assay

The StepOnePlus real-time polymerase chain reaction (PCR) system (Applied Biosystems, Foster City, CA, USA) was used for mRNA quantification. TaqMan™ Fast Advanced Master Mix (ThermoFisher, Germany) and commercially available Taqman primers and probes were utilized for both target genes. Each sample was run in triplicate and the results were analyzed using Sequence Detection software, version 1.9 (Applied Biosystems, Foster City, CA, USA). Gene expression for each signal was calculated using the difference-in-threshold-cycle procedure. The abundance of the target mRNA was quantified by calculating the differences in threshold cycles between the target genes. Standard curves for cDNA were generated using known concentrations of synthetic DNA oligonucleotides that have the same sequence as the corresponding target genes. These standards were serially diluted. Assays were accepted only if the R2 value for the standard curve was 0.97. Known sequences and concentrations of cDNAs were used as standards for each assay.

### Morphometric study of kidney biopsy

Renal scarring was studied using morphometry in previous studies [14, 15]. Specifically, renal biopsy specimens that were 5 μm thick underwent Jones’ silver staining. Semi-quantitative computerized image analysis was then performed using the Leica Twin Pro image analysis system from Leica Microsystems in Wetzlar, Germany. The analysis utilized MetaMorph 4.0 image-analyzing software from Universal Imaging Corporation in Downingtown, PA, USA. Each patient’s sample included ten glomeruli and ten randomly selected areas, which were evaluated to determine the average percentage of scarred glomerular and tubulointerstitial areas.

### Outcome measures

All patients were followed for a minimum of 12 months. The treatment decisions were made by their respective nephrologists and were not influenced by the study. Kidney function was regularly monitored, with assessments conducted at least every 3 months. The primary outcome measures focused on dialysis-free survival and renal event-free survival. The latter encompassed death from any cause, the need for dialysis, or a 40% decline in eGFR compared to the baseline. A secondary outcome measure involved calculating the rate of eGFR decline using the least-square regression method.

### Statistical analysis

The statistical analysis was conducted using SPSS for Windows software version 17.0 (SPSS, Chicago, IL). Results for normally distributed data were presented as mean ± SD, while skewed data were presented as median (inter-quartile range [IQR]). The Mann-Whitney U-test was used to compare gene expression levels between groups, and Spearman’s rank-order correlations were used to examine associations between gene expression levels and other parameters. Univariate Cox regression analysis was performed to analyze data for dialysis-free survival and renal event-free survival. Furthermore, a multi-variable Cox regression model was created, including age, sex, baseline eGFR, proteinuria, severity of glomerulosclerosis, and tubulointerstitial fibrosis. A statistically significant result was defined as a *P* value below 0.05, and all probabilities were two-tailed.

## Results

A total of 118 patients with type 2 diabetes and biopsied-proved DKD were recruited. Their duration of diabetes was 9.5 ± 4.1 years. We also studied 13 non-diabetic patients with biopsy-proved HTN as controls. Their baseline demographic and clinical characteristics are summarized and compared in Table 1.

**Table 1:** Baseline demographic and clinical data

	DKD	HTN	*P* value
no. of patients	118	13	
sex (M:F)	80:38	6:7	*p* < 0.0001 ^a^
age (years)	59.6 (53.5–66.7)	62.5 (51.3–70.9)	*p* = 0.852 ^b^
blood pressure (mmHg)			
systolic	138 (123–151)	129 (109–153)	
diastolic	76 (67–85)	72 (64–77)	
serum creatinine (µmol/l)	163 (122–255)	225 (103–414)	*p* = 0.143 ^b^
eGFR (ml/min/1.73m^2^)	37.7 (19.9–52.0)	24.9 (10.8–48.0)	*p* < 0.0001 ^b^
proteinuria (g/day)	2.5 (1.7–4.6)	0.6 (0.3–3.5)	*p* = 0.011^b^
CKD stage, no. of case (%)			*p* = 0.390
G1	5 (4.2%)	0	
G2	12 (10.2%)	2 (15.4%)	
G3a	26 922.0%)	1 (7.7%)	
G3b	28 (23.7%)	2 (15.4%)	
G4	27 (22.9%)	3 (23.1%)	
G5	20 (16.9%)	5 (38.5%)	
albuminuria stage, no. of case (%)			*p* < 0.0001
A1	11 (9.9%)	8 (61.5%)	
A2	59 (53.2%)	2 (15.4%)	
A3	48 (40.7%)	3 (23.1%)	
histological damage (%)			
glomerulosclerosis	30.0 (16.7–45.3)	27.8 (18.2–51.4)	*p* = 0.036 ^b^
tubulointerstitial fibrosis	30.0 (15.0–47.5)	25.0 (10.0–50.0)	*p* = 0.577 ^b^

DKD, diabetic kidney disease; HTN, hypertensive nephrosclerosis; eGFR, estimated glomerular filtration rate. Data are presented as median (inter-quartile range) and compared by ^a^Chi square test or ^b^Mann Whitney U test

### Urinary mRNA levels of podocyte stress markers

**Fig. 1:**
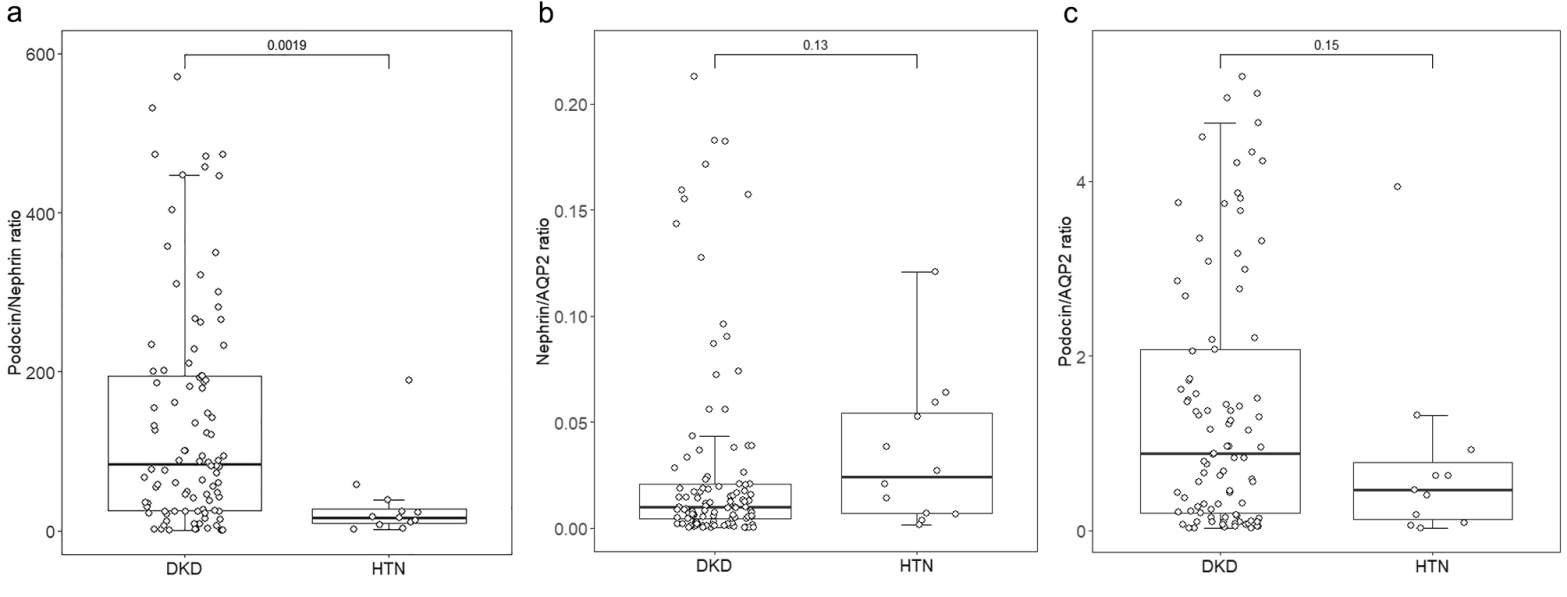
Comparison of urinary (**A**) podocin:nephrin ratio; (**B**) nephrin:AQP2 ratio; and (**C**) podocin:AQP2 ratio between patients with diabetic kidney disease (DKD) and hypertensive nephrosclerosis (HTN). Whisker-box plots, with boxes indicating median, 25th and 75th percentiles, whiskers indicating 5th and 95th percentiles. Data were compared by Mann-Whitney U test. (AQP2, aquaporin-2)

The urinary mRNA levels of nephrin, podocin, and AQP2 are summarized in Supplementary Table 1. The urinary podocyte stress marker levels of the DKD and HTN groups are summarized and compared in Fig. 1. In essence, urinary podocin:nephrin ratio of the DKD group was significantly higher than that of the HTN group (110.6 [IQR 34.9–328.4] vs. 17.6 [IQR 9.3–48.7], Mann Whitney U test, *p* = 0.0019), while there was no significant difference in urinary podocin:AQP2 ratio (1.40 [IQR 0.30–4.54] vs. 0.63 [IQR 0.13–2.63], *p* = 0.15) or nephrin:AQP2 ratio (0.012 [IQR 0.005–0.046] vs. 0.027 [IQR 0.007–0.062], *p* = 0.13) between the DKD and HTN groups.

### Relation with clinical and pathological parameters

The correlation between podocyte stress marker levels and clinical and pathological parameters of the DKD group are summarized in Table 2. In essence, there was a modest but significant correlation between urinary podocin:nephrin mRNA ratio and the degree of tubulointerstitial fibrosis (*r* = 0.254, *p* = 0.006), while urinary nephrin:AQP2 or podocin:AQP2 mRNA ratios did not correlate with any clinical or histological parameters.

**Table 2:** Relation Between urinary podocyte stress marker levels and clinical and pathological parameters

	podocin:nephrin ratio	nephrin:AQP2 ratio	podocin:AQP2 ratio
eGFR	*r* = -0.133, *p* = 0.151	*r* = 0.087, *p* = 0.347	*r* = -0.089, *p* = 0.305
CKD stage	*r* = 0.110, *p* = 0.235	*r* = 0.052, *p* = 0.573	*r* = -0.070, *p* = 0.454
proteinuria	*r* = -0.009, *p* = 0.925	*r* = -0.129, *p* = 0.180	*r* = -0.058, *p* = 0.517
albuminuria stage	*r* = 0.010, *p* = 0.911	*r* = -0.161, *p* = 0.082	*r* = -0.170, *p* = 0.065
glomerulosclerosis	*r* = 0.020, *p* = 0.827	*r* = 0.066, *p* = 0.479	*r* = 0.067, *p* = 0.445
tubulointerstitial fibrosis	*r* = 0.254, *p* = 0.006	*r* = -0.153, *p* = 0.101	*r* = 0.090, *p* = 0.302

eGFR, estimated glomerular filtration rate; AQP2, aquaporin-2

### Relation with clinical outcome

**Fig. 2:**
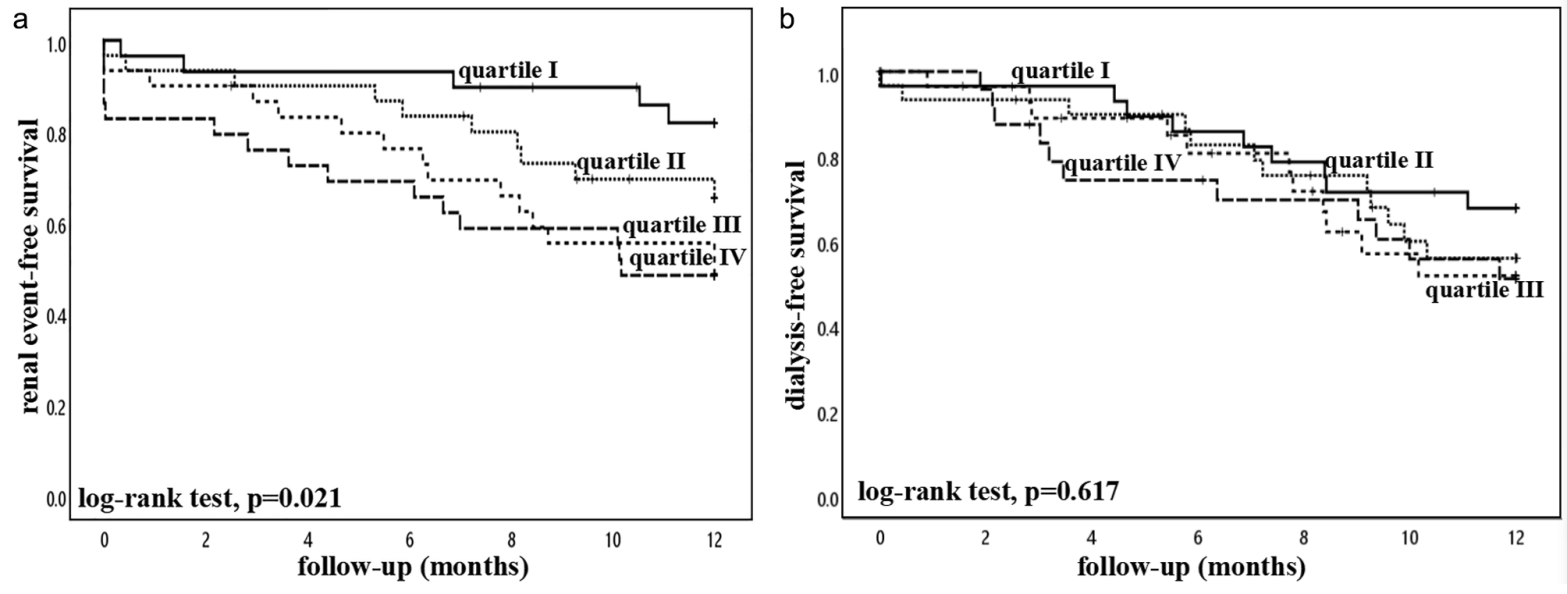
Kaplan-Meier plot of (**A**) renal event-free survival; and (**B**) dialysis-free survival of the diabetic kidney diseases group. Patients were divided according to the quartiles of urinary podocin:nephrin ratio, with quartile I indicating the lowest level. Data were compared with the log rank test

The DKD group were followed for up to 12 months. During the follow up period, none of the patients died; 36 patients progressed to dialysis-dependent kidney failure, and another 7 patients had 40% decline in eGFR. The 12-months renal event-free survival for urinary podocin:nephrin ratio quartile I to IV (with quartile I being the lowest levels) were 81.9%, 65.4%, 51.9%, 48.3%, respectively (log rank test, *p* = 0.021) (Fig. 2). The result of Cox regression analysis for the relation between podocyte stress marker level quartiles and renal event-free survival is further summarized in Table 3. Urinary podocin:nephrin ratio was associated with the renal event-free survival (unadjusted hazard ratio [HR], 1.523; 95% confidence interval [CI] 1.157–2.006; *p* = 0.003) by univariate Cox regression analysis. After adjusting the clinical parameters, urinary podocin:nephrin ratio have a trend to be an independent predictor of renal event-free survival (adjusted HR 1.327; 95%CI 0.980–1.797; *p* = 0.067), but the result did not reach statistical significance. In this model, baseline eGFR, age, and the severity of tubulointerstitial fibrosis were the independent predictors of renal event-free survival. None of the urinary podocyte stress marker was associated with dialysis-free survival or the rate of eGFR decline (Table 4).

**Table 3:** Cox regression model for renal event-free survival

	univariate analysis			multi-variable analysis		
	unadjusted HR	95%CI	*P* value	adjusted HR	95%CI	*P* value
sex	1.597	0.808–3.158	*p* = 0.178			
age	0.977	0.952–1.002	*p* = 0.071	0.961	0.930–0.993	*p* = 0.018
baseline eGFR	0.931	0.908–0.954	*p* < 0.001	0.934	0.907–0.961	*p* < 0.001
proteinuria	0.995	0.887–1.116	*p* = 0.927			
glomerulosclerosis	1.023	1.011–1.036	*p* < 0.001	0.991	0.977–1.005	*p* = 0.223
tubulointerstitial fibrosis	1.056	1.036–1.077	*p* < 0.001	1.035	1.009–1.061	*p* = 0.008
podocin:nephrin ratio quartile	1.523	1.157–2.006	*p* = 0.003	1.327	0.980–1.797	*p* = 0.067
nephrin:AQP2 ratio quartile	1.370	0.655–1.120	*p* = 0.161			
podocin:AQP2 ratio quartile	1.002	0.883–1.498	*p* = 0.152			

HR, hazard ratio; CI, confidence interval; eGFR, estimated glomerular filtration rate

**Table 4:** Relation between urinary podocyte stress marker levels and other outcome parameters

	dialysis-free survival^a^	slope of eGFR decline ^b^
podocin:nephrin ratio quartile	1.192 (0.911–1.558), *p* = 0.200	*r*=-0.224, *p* = 0.230
nephrin:AQP2 ratio quartile	1.308 (0.703–2.435), *p* = 0.397	*r* = 0.128, *p* = 0.535
podocin:AQP2 ratio quartile	1.003 (0.999–1.007), *p* = 0.122	*r*=-0.022, *p* = 0.917

eGFR, estimated glomerular filtration rate; AQP2, aquaporin-2

^a^unadjusted hazard ratio (95% confidence interval) by univariate Cox analysis; ^b^Spearman’s rank correlation coefficient

## Discussion

In our study, we found that urinary podocin:nephrin mRNA ratio correlated with the severity with tubulointerstitial fibrosis in patients biopsy-proved DKD and it showed a trend to predict renal event-free survival in this group of patients.

Podocyte injury plays an important role in the progression of diabetic kidney disease (DKD) [16]. Previous studies have suggested that urinary podocyte mRNA levels can precede microalbuminuria and predict the progression of diabetic nephropathy [17]. In a rat model of progressive kidney failure using human diphtheria toxin receptor (hDTR) transgenic rats, the urinary podocin:nephrin ratio was found to correlate with histologic damage [10]. Our results further confirm that the urinary podocin:nephrin mRNA ratio is significantly correlated with the severity of tubulointerstitial fibrosis in human DKD.

The original idea behind the urinary nephrin:AQP2 and podocin:AQP2 mRNA ratios, often referred to as “podocyte stress markers,” was to assess podocyte loss by detecting nephrin and podocin mRNA in urine. The use of AQP2 mRNA as a kidney reference gene helps account for variations in kidney contribution to RNA quantity and quality [7]. On the other hand, the podocin:nephrin mRNA ratio likely reflects qualitative changes in the podocyte, after adjusting for the number of podocytes (or their cellular fragments) lost in the urine [7]. Our results indicate that the qualitative alteration of podocytes serves as a prognostic marker for DKD (Diabetic Kidney Disease), while the contribution of podocyte damage in relation to other nephron segments is less significant.

Although we observed only a minor trend in the urinary podocin:nephrin mRNA ratio’s ability to predict renal events, we believe there is still significant potential for further developing this ratio as a biomarker for clinical use. The urinary podocin:nephrin mRNA ratio showed a significant correlation with the severity of tubulointerstitial fibrosis, but its independent prognostic value was diminished when included in the multi-variable model along with the latter. Since most patients with diabetic kidney disease (DKD) do not undergo kidney biopsy, it would be interesting to investigate whether the urinary podocin:nephrin mRNA ratio can predict the progression rate in these patients.

In this study, we also discovered that urinary AQP2 mRNA has a modest yet significant correlation with the severity of proteinuria in DKD. AQP2 is specifically expressed in the principal cells of renal collecting ducts [18]. Previous animal studies have shown that rosiglitazone can disrupt AQP2 regulation in diabetic mice, potentially leading to water retention after rosiglitazone treatment [19]. Another study has noted a close correlation between the abundance of urinary aquaporin-5 (AQP5) and the severity of DKD [20]. However, the significance of urinary AQP2, whether at the mRNA or protein level, has not been explored. Further investigation is warranted to study our findings regarding urinary AQP2 mRNA levels.

Our study has some limitations. Firstly, it was conducted at a single center and included DKD patients who underwent kidney biopsy. This may introduce referral bias, as patients with typical DKD are usually not referred for kidney biopsy. Secondly, we only measured the podocyte-associated mRNA level and did not assess the intra-renal level. Considering that podocin and nephrin are specific to podocytes, changes in their expression ratio indicate a modification in the inherent property of the podocyte. However, to further delineate the biological meaning of “podocyte stress”, further studies are needed to compare the levels of “podocyte stress” markers and objective assessment of podocyte density or integrity in the glomerulus. It would also be interesting to investigate whether a similar change is observed in the diabetic kidney. Lastly, the number of events was small, which hindered a comprehensive multivariable analysis.

In summary, our study showed that urinary podocin:nephrin mRNA ratio is a surrogate marker of the histological damage in biopsy-proved DKD and may be developed as a prognostic marker in this group of patients.

### Electronic supplementary material

Below is the link to the electronic supplementary material.

Supplementary Material 1

## Data Availability

The datasets used and/or analysed during the current study available from the corresponding author on reasonable request.
