# Supplementary material for: Urinary podocyte stress marker as a prognostic indicator for diabetic kidney disease
Source: BMC Nephrol. 2024 Jan 24;25:32. doi: 10.1186/s12882-024-03471-8 (PMC10807208; doi:10.1186/s12882-024-03471-8)
Supplement: Supplementary file 1 — Supplementary Material 1 [file 12882_2024_3471_MOESM1_ESM.docx]

# Supplementary Table 1. Absolute Levels of Urinary Podocyte Markers

|  | DKD | HTN | P value |
| --- | --- | --- | --- |
| nephrin mRNA level (copy/mmol-Cr ) | 7.38 (3.87-13.94) | 5.50 (2.95-9.27) | p = 0.182 |
| podocin mRNA level (copy/mmol-Cr) | 911.48 (225.80-3131.43) | 110.17 (51.97-339.94) | p = 0.019 |
| AQP2 mRNA level (copy/mmol-Cr) | 620.15 (165.22-2296.67) | 445.25 (1142.89-445.25) | p = 0.264 |

AQP2, aquaporin-2; DKD, diabetic kidney disease; HTN, hypertensive nephrosclerosis.

Data are presented as median (inter-quartile range) , and compared by Mann-Whitney U test.
